# Molecular subtyping of endometrial cancer via a simplified one-step NGS classifier, ARID1A and ZFHX4 mutations help further subclassify CNL/MSI-H patients

**DOI:** 10.1186/s13000-025-01652-z

**Published:** 2025-04-25

**Authors:** Qiuli Teng, Zeng Yuan, Yulong Mu, Xinyue Ma, Shuaixin Wang, Chenggong Sun, Linhan Chin, Zhan Huang, Changbin Zhu, Aijun Yin, Ruifen Dong

**Affiliations:** 1 Department of Obstetrics and Gynecology, Qilu Hospital, Shandong University, 107 West Wenhua Road, Ji’nan, Shandong 250012 People’s Republic of China; 2 Gynecology Oncology Key Laboratory, Qilu Hospital, Shandong University, Ji’nan, Shandong 250012 People’s Republic of China; 3 Department of Clinical Medicine, Medical School of Shandong University, Ji’nan, Shandong 250012 China; 4 Amoy Diagnostics Co., Ltd, No. 39, Dingshan Road, Haicang District, Xiamen, 361027 People’s Republic of China

**Keywords:** Endometrial carcinoma, Molecular classification, Next-generation sequencing, Prognosis, ARID1A, ZFHX4

## Abstract

**Background:**

Molecular subtyping has changed the prognostic stratification and therapeutic guidance for patients with endometrial cancer (EC). However, simultaneous application of sanger sequencing and immunohistochemistry under ProMisE criteria may be time- and tissue-consuming. This study attempted to measure subtype-specific biomarkers by one-step next-generation sequencing (NGS) resulting in a shorter turnaround time and less requirement of tissue samples.

**Methods:**

FFPE samples from 233 EC patients were retrospectively collected. Overall survival (OS) information was available for 131 patients with a median follow-up of 66 months. Genomic DNA was extracted and subjected to a one-step NGS panel including *TP53*, *POLE* and MSI measurement. Further comprehensive genomic analyses were performed on DNA from MSI-H and copy number low (CNL) subtypes.

**Results:**

The molecular typing ratio of the 233 patients was 8.15% for *POLE* subtype, 18.88% for MSI-H subtype, 11.59% for copy number high (CNH) subtype and 61.37% for CNL subtype. The 10-year OS and disease-specific survival (DSS) rate was 100% in *POLE* subtype, while only 33.51% and 39.69% in CNH subtype. In patients with CNL and CNL/MSI-H subtypes, *ARID1A* and *ZFHX4* mutations were significantly associated with worse prognosis respectively.

**Conclusion:**

This simplified one-step NGS panel can effectively subgroup EC patients into four prognostically different subtypes. New biomarkers are able to potentially refine the classification of patients with CNL/MSI-H subtypes into groups with distinct clinical outcomes.

**Supplementary Information:**

The online version contains supplementary material available at 10.1186/s13000-025-01652-z.

## Background

Endometrial carcinoma (EC) is one of the three most common gynecologic malignancy with a rising incidence and mortality rate, and the average age of onset is becoming younger globally [1, 2]. Previously, the diagnosis and staging of EC relied mainly on clinical and histopathological findings. However, ECs are a heterogenous group of tumors that exhibit different histological structures, molecular abnormalities, and clinical outcomes [3]. The heterogeneous nature of EC makes a significant limitation to rely on limited histopathology to assess the disease. With increasing research on molecular biology, a new classification of EC, molecular typing [4], has been proposed and widely accepted in clinical practice.

In 2013, The Cancer Genome Atlas (TCGA) identified four distinct prognostic subtypes of EC based on a combination of whole genome or exome sequencing, microsatellite instability (MSI) assays, and copy number analysis using array- and sequencing-based technologies: *POLE* (ultra-mutated); MSI-high (MSI-H, hypermutated); copy number high (CNH, serous-like); and copy number low (CNL, endometrioid) [5]. TCGA molecular typing has important clinical advantages in studying clinical outcomes of EC patients, improving prevention and treatment methods, and identifying cancer immunotherapy beneficiaries. However, this method is not easy to be applied in clinical practice because of the high cost of gene sequencing and long waiting time for results, and the classification method is not applicable to specimens such as biopsies/scrapings, which cannot accurately guide patients’ preoperative treatment.

Inspired by these findings, A Talhouk et al. developed a simplified classifier, termed Proactive Molecular Risk Classifier for Endometrial Cancer (ProMisE), which identifies four molecular subtypes that are analogous but not identical to the four genomic subtypes described in TCGA: *POLE* exonuclease domain mutated (POLE EDM), mismatch repair deficient (MMRd), p53 wild-type/CNL (p53wt), and p53-mutated/CNH (p53abn) [6]. Subsequently, the TransPORTEC international consortium identified four very similar molecular subtypes with distinct outcomes and high diagnostic reproducibility [4]. Both the ProMisE classifier and the TransPORTEC classification utilized similar core molecular signatures and classified patients using a combination of immunohistochemical (IHC) and next-generation sequencing (NGS) [7]. The advantages of molecular typing based on IHC approach lie in its easy to obtain, low cost, and short in cycle time. However, the current IHC-only approach cannot yet replace *POLE* mutation sequencing results. Therefore, NGS detection of *POLE* gene is still required as an important part of ProMisE classifier, and the need for multi-technical cooperation is also one of the major obstacles in the integration of ProMisE classifier into clinical diagnosis.

Recently, studies attempted to realize molecular typing of EC by detecting MSI status and *TP53* mutations only by NGS, and the consistency with traditional ProMisE typing was as high as 98.1% (52/53, kappa = 0.97) [8, 9]. The detection of MSI and *TP53* mutations by NGS may be an alternative to the detection of MMR and p53 expression by IHC, however, further studies are still needed for verification.

Most ECs are classified as p53wt and no specific molecular features (NSMP) in the ProMisE and TransPORTEC classification, respectively [6, 10]. Although patients with p53wt/NSMP subtype usually have a stable genome, low level of somatic copy number alterations, and a moderate prognosis [10], they were found to be rather heterogeneous and there is an urgent need to find new risk stratification biomarkers to better guide their clinical management [11]. Such as mutations in CTNNB1 exon 3, which were associated with poor prognosis in NSMP EC [12].

Given the existence of the aforementioned clinical issues, in the present study, an all-in one simplified NGS panel covering *POLE*, *TP53* and MSI was used to develop an NGS-based classifier. The effectiveness and feasibility of the clinical application of using NGS test for EC typing was then evaluated by comparison with the existing methods. In addition, we analyzed the relationship between potential markers detected by NGS and prognosis of CNL patients.

## Methods

### Study design

**Fig. 1:**
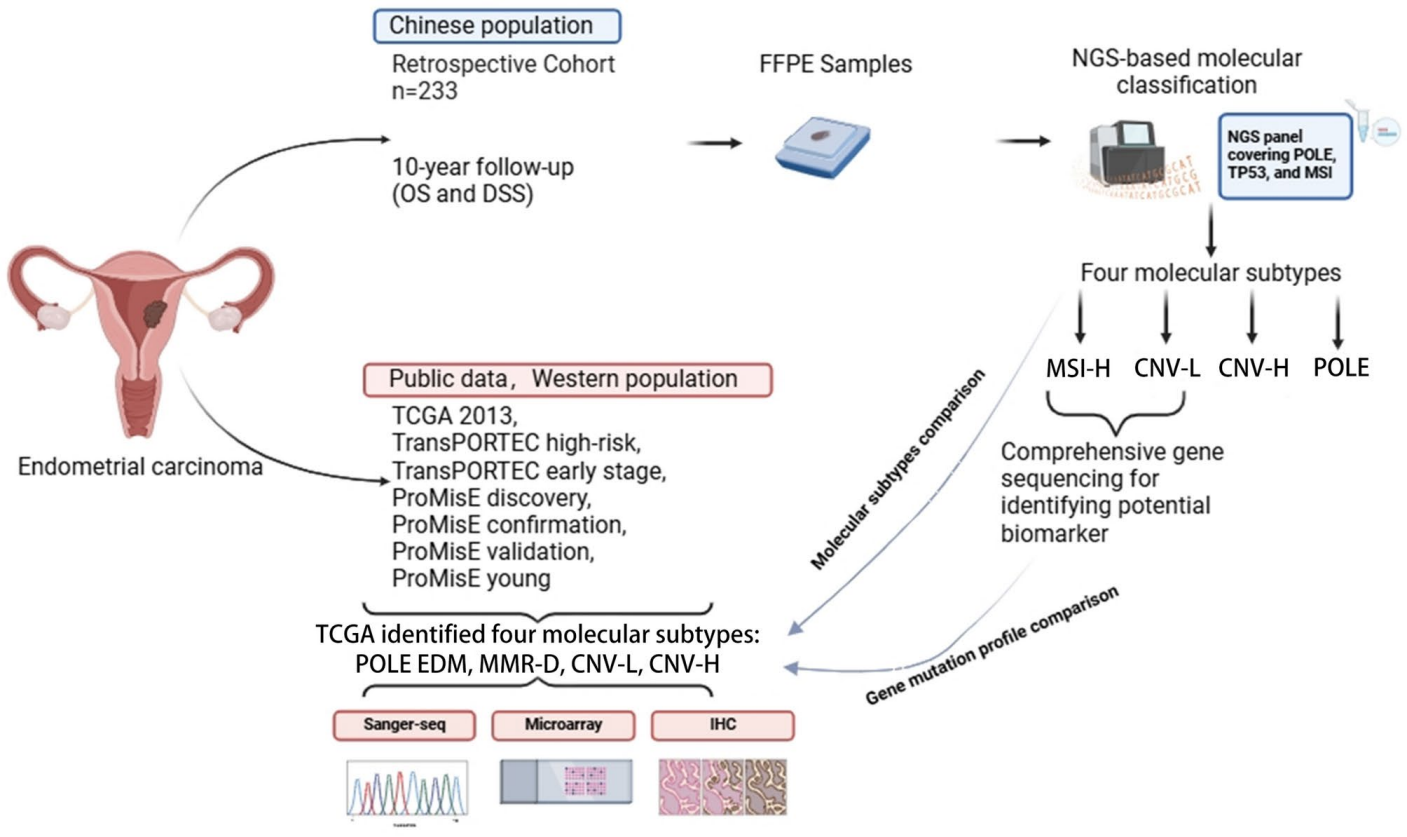
Study design

Formalin-fixed paraffin-embedded (FFPE) samples from 233 patients with cytologically or histologically confirmed EC were retrospectively collected at Qilu Hospital, Shandong Province between 2017 and 2018 were collected. Demographic data such as age (range, 30–84 years) and family history, as well as clinical treatment information including histopathological subtypes, tumor stage, treatment strategies, disease-specific survival (DSS) and overall survival (OS) were obtained from hospital records and clinical trial center of Qilu Hospital. Patients were staged according to the International Federation of Gynecology and Obstetrics (FIGO) criteria. The collected samples were all tested for molecular typing, and the clinicopathological characteristics and prognostic features of each molecular subtype were analyzed. And some of the MSI-H and CNL subtype samples were screened for multi-driven gene variants testing to explore molecular variant characteristics associated with prognosis. Data from public database were also included for comparison with local assay data in attempt to assess molecular classification differences in EC among different ethnic groups (Fig. 1). This study was done in accordance with the principles of the Declaration of Helsinki. The study protocols were approved by the ethical committee of Shandong University (KYLL-202210-055-1). All patients provided written informed consent.

### DNA extraction, library construction and targeted sequencing for molecular classification

An all-in-one NGS Panel (Amoy Diagnostics, Xiamen, China) covering *POLE*, *TP53*, and MSI was used for sequencing. The panel can comprehensively and accurately detect single nucleotide variants (SNVs), insertions and deletions (InDels), and hotspot mutations of oncogenic genes. Total genomic DNA was extracted from FFPE tumor tissue using the FFPE DNA kit (Amoy Diagnostics, Xiamen, China) following the manufacturer’s instructions. DNA libraries were constructed and the probes were captured using the BPTM detected kit (Amoy Diagnostics, Xiamen, China) after quality control. The captured DNA libraries were measured with a QuantiFluor dsDNA System (Promega, Madison, Wisconsin, USA), and library insertion fragment size was detected using the Agilent 2100 (Agilent Technologies, Santa Clara, California, USA). After library quality control, 2 × 150 bp paired end sequencing of the prepared libraries was performed using the NovaSeq 6000 system (San Diego, California, USA). Sequence data was analyzed on the AmoyDx NGS Data Analysis System (ANDAS, Amoy Diagnostics, Xiamen, China) for accurate detection of SNVs, InDels with a sensitivity of ≥ 5% of variant allele frequency (VAF).

After removing adaptor and low-quality reads, clean FASTQ data were aligned to the human reference genome (hg19) using the BWA-MEM aligner with default parameters (http://biobwa.sourceforge.net/). UMI was used for analyzing the quality control indicators including sequencing depth, alignment rate, coverage rate and uniformity of the processed data. The subtypes of tumor samples (POLE, MSI-H, CNH, or CNL) were determined through the following hierarchical classification process: First, samples were classified as the POLE subtype if a pathogenic *POLE* gene variant (including ultramutated status) was detected based on genetic testing results. Second, if the *POLE* gene was wild-type or harbored a non-pathogenic variant, the microsatellite instability (MSI) status was assessed. Samples exhibiting hypermutation in the context of MSI were classified as the MSI-H subtype. Third, if the MSI status was MSS (with MSI-L and MSS collectively classified as MSS), samples were categorized into CNH and CNL obtained from the in-house algorithm developed by NGS.

### Targeted sequencing of multi-driver gene mutation

A customized 571-gene panel (Amoy Diagnostics, Xiamen, China) was used in the present study. The detection processes described in the manufacture’s instruction were followed. For NGS sequencing, 150ng DNA was used for fragmentation and library construction. After the quality check, 500ng library DNA was taken for hybridization capture, and the concentration of the library should reach 2.5 ng/µL after capture. Paired-end sequencing (2 × 150 bp) was performed on Illumina NovaSeq 6000, and 8 Gb of sequencing data was obtained. AmoyDx NGS data analysis system-ANDAS Data analyzer was used for sequencing data analysis to obtain the variation information of target genes. The detection sensitivity of hotspot variants and non-hotspot variants was ≥ 1% VAF and ≥ 5% VAF, respectively.

### The public data

The EC molecular classification data and clinical metadata were obtained from public reports, including TCGA 2013 [13], TransPORTEC high-risk [10], TransPORTEC early stage [14], ProMisE discovery [6], ProMisE confirmation [15], ProMisE validation [16], ProMisE young [17]. The proportions of the four molecular subtypes were compared in the present study as well as the aforementioned public data. The clinicopathological differences among the four molecular subtypes, as well as *POLE* and *TP53* mutation profiles, and associated clinicopathological characteristics, were also compared between the present data and the TCGA 2013.

### Statistical analysis

Differences of clinicopathological characteristics between the indicated groups were compared using the t-test or one-way analysis of variance (ANOVA) followed by Fisher’s least significant difference (LSD) test, appropriately. Kaplan–Meier estimations with log-rank test were performed to compare the significance of prognostic differences among different molecular subtypes. The level of concordance of molecular profile in both classifiers was determined using overall accuracy and Cohen’s kappa estimates. 95% confidence intervals (CI) were calculated using the bootstrap method with 1000 bootstrap samples. Cox multivariate regression analysis was used to analyze pathological factors affecting OS. A two-sided *p* < 0.05 was considered statistically significant. SPSS, RRID: SCR_002865 (IBM Corp., Armonk, NY, USA) was used for statistical analyses.

## Results

### Patient characteristics

A total of 233 ECs were available for analysis (Fig. 1). The demographic and histopathological characteristics of all cases are summarized in Table 1. The median age of the patient was 55 years (range, 30–84). Compared to the pathological characteristics of patients in the TCGA 2013 cohort, patients in the present cohort were characterized by younger age (54.79 vs. 63.14, *p* < 0.01), highly differentiated (G1, 41.63% vs. 29.31%, *p* < 0.01), lower proportion of grade 3 (G3, 19.31% vs. 39.66%, *p* < 0.01), and lower proportion of serous carcinomas (4.72% vs. 18.10%, *p* = 0.01). There were 157 (67.38%) patients diagnosed with FIGO stage I disease, and of the remaining cases, 17 (7.30%) were stage II, 34 (14.59%) were stage III, 3 (1.29%) were stage IV, and 22 (9.44%) with unknow stage. Lympho-vascular space invasion (LVSI) was evaluable in 186 cases of which 13 (5.58%) were found to be positive. The majority of cases (183/78.54%) were endometrioid histotype, 11 (4.72%) were serous, 2 (0.86%) were mucinous, the rest were mixed histology and unknow type. Grade distribution was evaluable in 197 cases, including 97 (41.63%) grade 1 (G1), 55 (23.61%) grade 2 (G2), and 45 (19.31%) grade 3 (G3).

**Table 1:** Demographic and histopathological characteristics of EC patients: the present cohort and TCGA 2013

	Total			Typing of the present cohort based on NGS detection				
Items/*N* (%)	TCGA 2013 cohort (*N* = 232)	The present cohort (*N* = 233)	*p* value	POLE (*N* = 19)	MSI-H (*N* = 44)	CNH (*N* = 27)	CNL (*N* = 143)	*p* value
**Age (number)**			< 0.01					0.04
Mean ± SD	63.14 ± 10.94	54.79 ± 9.82		53.65 ± 10.21	56.32 ± 8.93	59.33 ± 10.88	53.63 ± 9.62	
Median [min-max]	63.00 [33.00, 90.00]	55.00 [30.00, 84.00]		57.00 [30.00, 68.00]	56.50 [36.00, 75.00]	61.50 [39.00, 84.00]	54.00 [31.00, 76.00]	
**Stage**			0.36					0.15
I	166 (71.55%)	157 (67.38%)		16 (84.21%)	26 (59.09%)	12 (44.44%)	103 (72.03%)	
II	12 (5.17%)	17 (7.30%)		0 (00.00%)	6 (13.64%)	3 (11.11%)	8 (5.59%)	
III	41 (17.67%)	34 (14.59%)		2 (10.53%)	6 (13.64%)	8 (29.63%)	18 (12.59%)	
IV	11 (4.74%)	3 (1.29%)		0 (00.00%)	0 (00.00%)	1 (3.70%)	2 (1.40%)	
Unknow	2 (0.86%)	22 (9.44%)		1 (5.26%)	6 (13.64%)	3 (11.11%)	12 (8.39%)	
**LVSI**								0.07
No		173 (74.25%)		13 (68.42%)	33 (75.00%)	18 (66.67%)	109 (76.22%)	
Yes		13 (5.58%)		2 (10.53%)	1 (2.27%)	5 (18.52%)	5 (3.50%)	
Unknow		47 (20.17%)		4 (21.05%)	10 (22.73%)	4 (14.81%)	29 (20.28%)	
**Histology Subtype**			0.01					< 0.01
Endometrioid	186 (80.17%)	183 (78.54%)		15 (78.95%)	34 (77.27%)	15 (55.56%)	119 (83.22%)	
Mucinous		2 (0.86%)		0 (00.00%)	2 (4.55%)	0 (00.00%)	0 (00.00%)	
Serous	42 (18.10%)	11 (4.72%)		2 (10.53%)	0 (00.00%)	6 (22.22%)	3 (2.10%)	
Others	4 (1.72%)	7 (3.00%)		0 (00.00%)	2 (4.55%)	4 (14.81%)	1 (0.70%)	
Unknow		30 (12.88%)		2 (10.53%)	6 (13.64%)	2 (7.41%)	20 (13.99%)	
**Grade**			< 0.01					< 0.001
G1	68 (29.31%)	97 (41.63%)		9 (47.37%)	12 (27.27%)	4 (14.81%)	72 (50.35%)	
G2	72 (31.03%)	55 (23.61%)		2 (10.53%)	13 (29.55%)	5 (18.52%)	35 (24.48%)	
G3	92 (39.66%)	45 (19.31%)		6 (31.58%)	11 (25.00%)	14 (51.85%)	14 (9.79%)	
Undifferentiated		3 (1.29%)		0 (00.00%)	1 (2.27%)	1 (3.70%)	1 (0.70%)	
Unknow		33 (14.16%)		2 (10.53%)	7 (15.91%)	3 (11.11%)	21 (14.69%)	

### Molecular typing performed through the simplified NGS panel in the present EC patient cohort

**Fig. 2:**
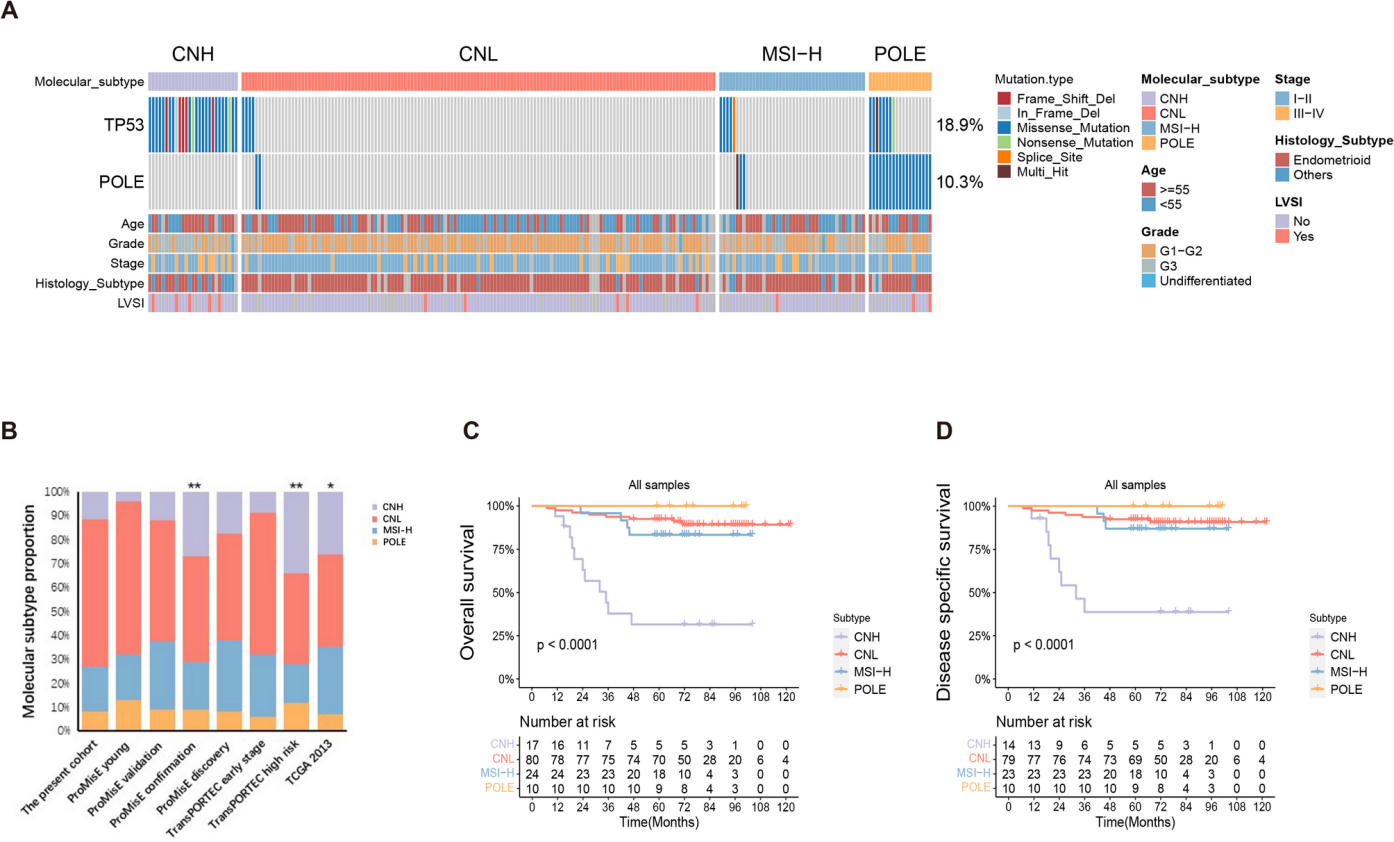
Overview of molecular classification of the enrolled EC patients. **(A)** Landscape of mutation profiles as well as the molecular subtypes in the present cohort. **(B)** The proportion of molecular typing was compared between the present study and the previous data. The 10-year overall **(C)** and disease-specific survival **(D)** of the four molecular subtypes

The molecular typing of 233 EC tissue samples was performed using the simplified NGS panel. The proportion of POLE (*n* = 19), MSI-H (*n* = 44), CNH (*n* = 27) and CNL (*n* = 143) subtypes were 8.15%, 18.88%, 11.59%, and 61.37%, respectively (Fig. 2A). We analyzed the distributions of the four subtypes in the different pathological subtypes (Table 1; Fig. 2A). The results showed that the molecular subtypes were significantly associated with age (*p* = 0.04), grade (*p* < 0.001) and histology (*p* < 0.01). POLE subtype was enriched in G1 (47.37%), G3 (31.58%), and endometrioid histotype (78.95%). CNH subtype was enriched in older age (59.33 ± 10.88 years), G3 (51.85%), endometrioid (55.56%) and serous (22.22%) disease. Although there were no statistically significant differences in FIGO stage among the four molecular subtypes, the proportion of patients with stage I was highest in the POLE subtype (84.21%), followed by the CNL subtype (72.03%), and the proportion of patients with stage IV was highest in the CNH subtype (3.70%). The distribution of LVSI among the four subtypes was 2 (0.86%), 1 (0.43%), 5 (2.15%) and 5 (2.15%) in POLE, MSI-H, CNH and CNL subtype, respectively.

### The NGS-based subtyping was significantly associated with patient’s prognosis

The subtype distribution of this cohort was compared with that of previous cohorts. As shown in Fig. 2B, the proportions of the four molecular subtypes in this cohort were not significantly different from other previous cohorts, except for the ProMisE confirmation cohort (*p* < 0.01), the TransPORTEC high-risk cohort (*p* = 0.001), and the TCGA 2013 cohort (*p* = 0.02). However, there were no significant differences in the mutation profiles of *POLE* and *TP53* between the present cohort and the TCGA 2013 cohort (Supplementary Fig. 1A-B, and Supplementary Tables 1–2). The prognosis value of NGS-based molecular classification in the present cohort was evaluated by comparing their 10-year OS and DSS. OS data were obtained from 131 patients with NGS classification results, with a median follow-up of 66 (7-122) months, of whom 126 patients had DSS data. 10-year OS rate (*p* < 0.001) and DSS rate (*p* < 0.001) of the four molecular subtypes were statistically different. 10-year OS and DSS rate of the POLE subtype were 100%, respectively, while the 10-year OS and DSS rate of the CNH subtype were only 33.51% and 38.69%. The median OS and median DSS of the CNH subtype were 35 months and 32 months, respectively, while the other three subtypes were not reached (Fig. 2C-D). Outcomes by molecular subtype were consistent with what was previously observed.

**Fig. 3:**
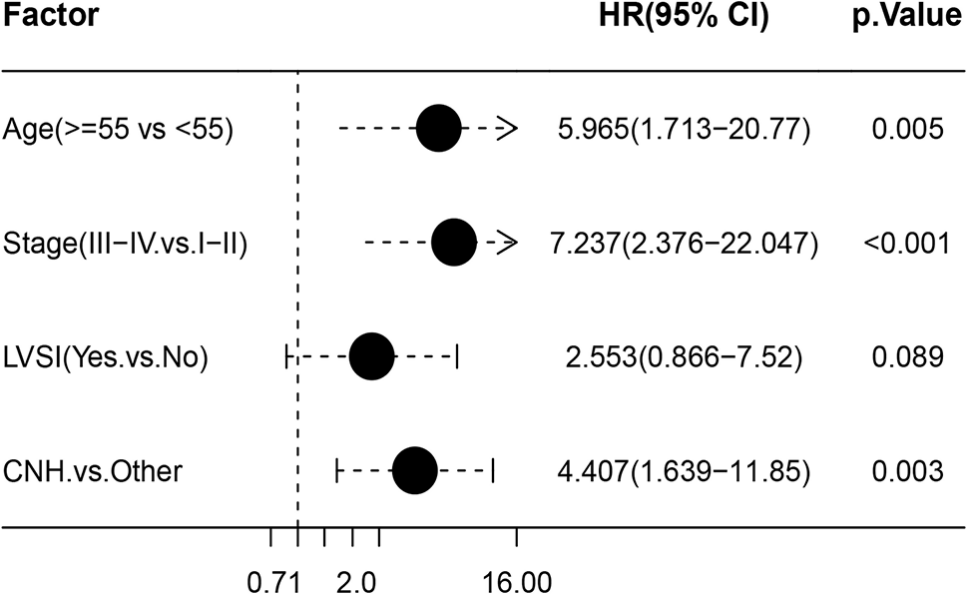
Factors significantly associated with 10-year overall survival was analyzed by multivariate Cox regression model

Among clinicopathologic features and molecular classification, multivariate Cox regression analysis showed age, FIGO stage, and molecular subtypes to be significant associated with prognosis (Fig. 3). Age ≥ 55 was accompanied with a Hazard Ratio (HR) of 5.965 (95% CI: 1.713–20.77) for OS, later stage was accompanied with a HR of 7.237 (95% CI: 2.376–22.047) for OS, and CNH subtype was accompanied with a HR of 4.407 (95% CI: 1.639–11.85) for OS. Here, we identified that LVSI positive was at higher risk for fatal outcome but with a non-statistically significant (HR: 2.553, 95% CI: 0.866–7.52, *p* = 0.089, Fig. 3).

We further stratified outcomes of patients across molecular subtypes according to grade, stage, LVSI and age. Outcomes of molecular subtypes (OS) were still statistically different in patients with G1/G2 disease (*p* < 0.01), stage III-IV (*p* = 0.03), LVSI negative (*p* < 0.001), and age ≥ 55 (*p* < 0.001) (Supplementary Fig. 2).

### Prognostic biomarkers exploration in patients with CNL and MSI-H subtypes

A total of 39 specimens from 27 patients with CNL subtype and 12 patients with MSI-H subtype were tested using a customized 571-gene NGS panel. The clinicopathological features of these patients were not significantly different from those of all patients with CNL and MSI-H subtypes, except for a higher proportion of G3 patients (*p* < 0.01) (Table 2).

**Table 2:** Clinicopathological features of patients with MSI-H and CNL subtypes

Items/*N* (%)	All patients in MSI-H and CNL subtypes (*N* = 187)	Patients with comprehensive genetic testing in MSI-H and CNL subtypes (*N* = 39)	*p* value
**Subtype**			0.45
CNL	143 (76.47%)	27 (69.23%)	
MSI-H	44 (23.53%)	12 (30.77%)	
**Age (number)**			0.24
Mean ± SD	54.25 ± 9.51	56.54 ± 8.86	
Median [min-max]	54.00 [31.00, 76.00]	59.00 [31.00, 75.00]	
**Stage**			0.21
I	129 (68.98%)	28 (71.79%)	
II	14 (7.49%)	1 (2.56%)	
III	24 (12.83%)	9 (23.08%)	
IV	2 (1.07%)	0 (0.00%)	
Unknow	18 (9.63%)	1 (2.56%)	
**LVSI**			0.08
Yes	6 (3.21%)	3 (7.69%)	
No	142 (75.94%)	33 (84.62%)	
Unknown	39 (20.86%)	3 (7.69%)	
**Histology Subtype**			0.11
Endometrioid	153 (81.82%)	38 (97.44%)	
Serous	3 (1.60%)	1 (2.56%)	
others	5 (2.67%)	0 (0.00%)	
Unknown	26 (13.90%)	0 (0.00%)	
**Grade**			< 0.01
G1	84 (44.92%)	10 (25.64%)	
G2	48 (25.67%)	16 (41.03%)	
G3	25 (13.37%)	12 (30.77%)	
Undifferentiated	2 (1.07%)	1 (2.56%)	
Unknown	28 (14.97%)	0 (0.00%)	
**OS**			0.75
Mean ± SD	66.18 ± 24.35	75.21 ± 31.23	
Median [min-max]	67.00 [7.00, 122.00]	74.00 [7.00, 121.00]	

**Fig. 4:**
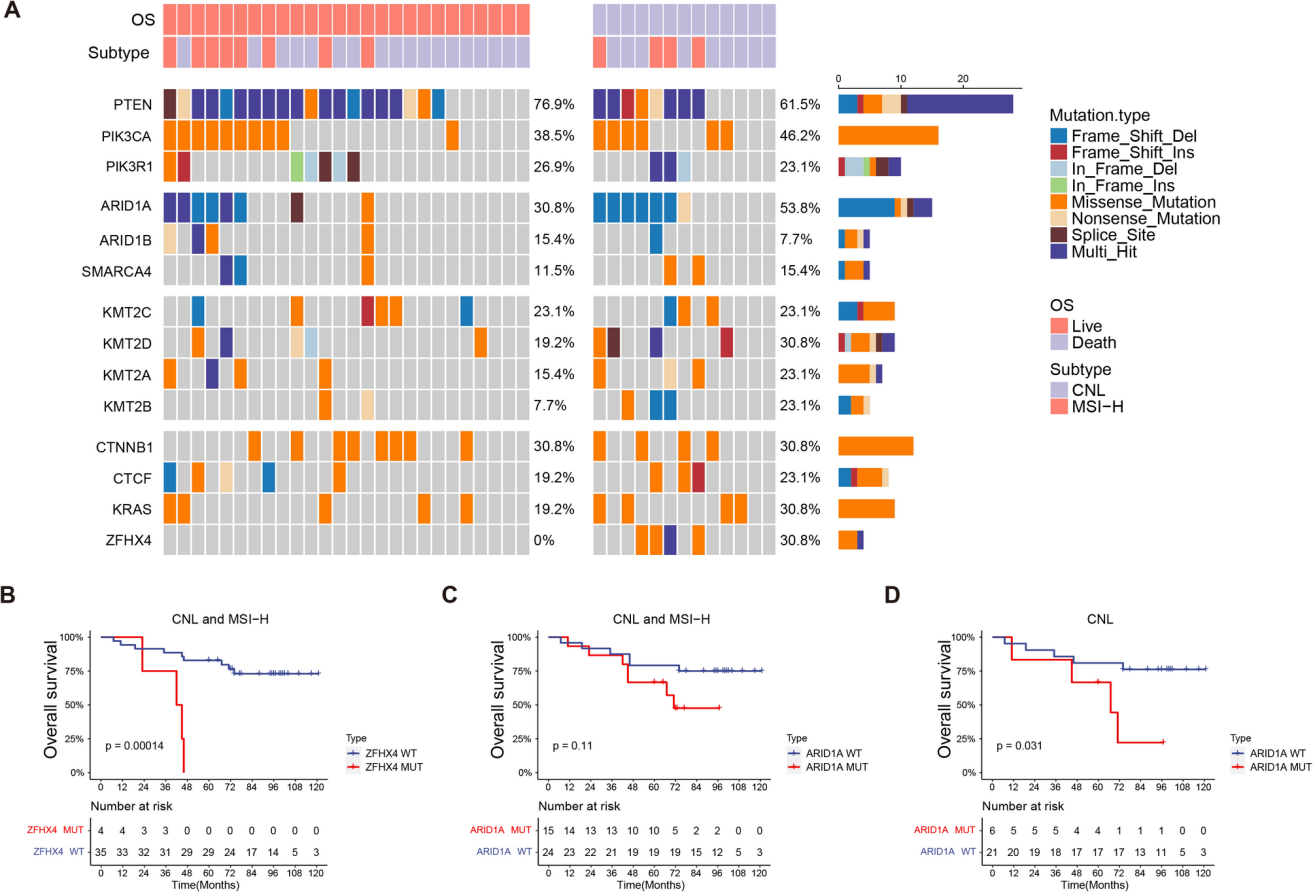
Prognostics biomarker analysis of CNL and MSI-H carcinomas. **(A)** Driver gene mutation profile of CNL and MSI-H subtype was shown in the waterfall plot. Overall survival analysis of MSI-H and CNL carcinomas with or without *ZFHX4***(B)**, *ARID1A***(C)** mutation, and overall survival analysis of CNL carcinomas with or without *ARID1A***(D)** mutation

The 571-gene NGS panel sequencing results of 39 CNL/MSI-H samples showed that mutations were mainly concentrated in the mTOR signaling pathway, SWI/SNF complex, H3K4 methyltransferase, and *CTNNB1*, *CTCF*, *KRAS*, and *ZFHX4*. The aforementioned gene mutation frequencies were generally higher in deceased cases than in surviving cases, with *ARID1A* (53.8% vs. 30.8%), and *ZFHX4* (30.8% vs. 0%) being the most representative (Fig. 4A). The *ARID1A* gene mutations detected in this study were partially consistent with the TCGA 2013 cohort. However, mutations in the *ZFHX4* gene were not detected in the TCGA 2013 cohort (Supplementary Fig. 1C-D). Kaplan–Meier analyses showed significantly worse outcome for *ZFHX4* mutant, CNL/MSI-H tumors with 10-year OS rates of 0% for *ZFHX4* mutant and 73.0% for *ZFHX4* wt tumors (*p* < 0.001, Fig. 4B). For CNL/MSI-H tumors with *ARID1A* mutations, only a prognostic trend for *ARID1A* mutation and worse outcome was observed (*p* = 0.11, Fig. 4C). However, *ARID1A* mutation was significantly associated with worse prognosis in TP53wt cases (*p* = 0.03, Fig. 4D).

## Discussion

The molecular classification of EC using either the TCGA classification method or the ProMisE or TransPORTEC methods has the clinical problem of being time and tissue-consuming. Currently, several studies are devoted to using simpler and more tissue-sparing methods, such as NGS technique, to classify EC patients, and it has been found that NGS and IHC provide equal information for molecular subtype determination [8, 9]. In this study, we investigated the distribution of molecular subtypes in Chinese EC patients using a one-step, simplified NGS panel, and evaluated the differences in clinicopathological characteristics among the subtypes. Our results indicated that the four subtypes (POLE, 8.15%; MSI-H, 18.88%; CNH, 11.59%; and CNL, 61.37%) classified based on NGS technology were significantly associated with patient prognosis. Compared with the TCGA 2013 cohort, the higher proportion of *TP53*wt subtype and lower proportion of *TP53* mutant subtype in the present cohort were speculated to be due to the younger patients enrolled in this study and fewer patients with high pathological risk (G3 and serous carcinomas) [13]. Consistent with the reported data [13, 16, 18], this study also found that patients with CNH subtype were older, and had a higher proportion of patients with G3, stage III-IV, serous carcinomas, and LVSI positive, which predicted a poor prognosis for patients with CNH subtype. However, it was shown that there were a few patients with high-risk pathological features in POLE subtypes, such as 6 out of 45 G3 patients, 2 out of 11 serous carcinoma patients, and 2 out of 13 LVSI positive patients were POLE subtypes. Therefore, more accurate tumor molecular classification of EC patients should be performed clinically to predict survival and response to treatment.

Previous studies have demonstrated that EC with POLE ultramutated and MSI-H profiles exhibit an extremely high mutational burden, which predisposes these tumors to acquire numerous secondary mutations, including those in *TP53* [5, 6]. In these contexts, *TP53* mutations are frequently observed but are generally regarded as passenger mutations—occurring because of the hypermutated genomic landscape rather than as primary oncogenic drivers. In contrast, *TP53* mutations in CNH or serous-like tumors are typically early, driver events that contribute directly to tumor development and progression, correlating with more aggressive clinical behavior and poorer outcomes [19]. The differential biological significance of *TP53* mutations is thus highlighted by their distinct roles: while the presence of *TP53* mutations in CNH tumors is associated with a dismal prognosis, similar mutations in POLE ultramutated or MSI-H tumors do not seem to exert an equivalent impact on clinical outcomes. This underscores the importance of integrating molecular context into the interpretation of *TP53* alterations in EC.

Our study demonstrated a significant correlation between molecular classification and clinical outcome in the present EC cohort. In detail: patients with POLE subtype had the best survival (OS and DSS), while patients with CNH subtype had the worst prognosis, which was consistent with previous studies [13, 16, 18]. Furthermore, similar to the previous study [20], age, FIGO stage, and molecular typing were found to be correlated with the prognosis of EC patients in our cohort. Further stratification of each molecular subtype using these clinical characteristics revealed that outcomes of molecular subtypes were statistically different in patients with G1/G2 disease, stage III-IV, LVSI negative, and age ≥ 55. Therefore, our simplified NGS-based EC molecular classifier was able to effectively classify EC patients into four prognostically significant subtypes.

Both this study and previous reports indicated that the majority of EC (30–60%) exhibits TP53wt (CNL)/NSMP subtype [21], and this subtype was found to be rather heterogeneous. Therefore, in this study, partial samples of the CNL subtype and another subtype with a relatively high proportion, MSI-H subtype, were further selected for multigene sequencing, hoping to find prognostic biomarkers for stratification of these patients. Gene sequencing results showed a high frequency of mutations in mTOR signaling pathway genes: *PTEN*, *PIK3CA*, *PIK3R1*, and SWI/SNF complex unit genes *ARID1A*, which was consistent with previous reports [22–24]. In addition, the major component genes of the H3K4 methyltransferase were detected with high frequency in these specimens, particularly concentrated in MSI-H samples, a feature that has also been reported in a pan-cancer study [25]. Loss of the *ARID1A* gene has been reported to be associated with poor prognosis in a variety of tumors [26–28], and a study on EC published in 2021 also showed that TP53wt/NSMP carcinomas harboring *ARID1A* mutations were more likely to relapse [29]. In the present study, *ARID1A* mutation was also associated with poor prognosis in CNL carcinomas. In addition, four *ZFHX4* mutations were identified here, three in MSI-H subtype and one in CNL subtype, all of which occurred in deceased cases. The ZFHX4 protein is a key molecular regulator of tumor-initiating stem cell-like function. Previous analysis of public data showed that *ZFHX4* gene mutation was an independent prognostic factor of poor outcome in ovarian cancer [30, 31]. Our study demonstrated that EC tumors with the *ZFHX4* mutation in the CNL and MSI-H subtypes had a significantly worse prognosis.

*ARID1A* and *ZFHX4* mutations emerged as independent negative prognostic factors in the CNL and CNL/MSI-H subgroups. *ARID1A* encodes a component of the SWI/SNF chromatin remodeling complex and is frequently mutated in endometrioid and *POLE*-ultramutated tumors. While *ARID1A* loss in *POLE*-ultramutated tumors may be mitigated by the strong immunogenicity of that subtype, its presence in non-*POLE* tumors may confer increased risk through mechanisms such as impaired DNA repair and immune evasion. *ZFHX4*, a transcription factor implicated in chromatin dynamics, has been associated with increased genomic instability in other tumor types and may similarly contribute to aggressive tumor behavior in endometrial cancer. These findings suggest that *ARID1A* and *ZFHX4* mutation status could aid in refining risk stratification within otherwise intermediate-risk molecular categories.

Although the classifier based on this one-step simplified NGS panel can effectively classify Chinese EC patients into four prognostic subtypes, our study still has certain limitations. First, the follow-up time in this study was insufficient, resulting in the median OS not being achieved in the other three subtypes except the CNH subtype; however, we will continue to follow up these enrolled patients. Second, comparing the concordance between NGS-based and IHC-based typing methods is a better way to determine whether NGS typing methods are manageable in clinical practice, as IHC has been used as the gold standard for detecting biomarkers in clinical [21, 32]. However, due to the incomplete information in this retrospective research, our study was not able to compare the consistency. In further studies, test results from different techniques will continue to be collected for methodological comparison. Third, the number of samples available for comprehensive genetic testing was limited, so future works are needed to validate the biomarkers identified in this study that can be used to stratify patients with the CNL subtype. This study validated that the one-step NGS-based molecular classification can effectively and clinically practicable to classify Chinese EC patients into four prognostically associated subtypes. Furthermore, putatively stratification of patients with CNL/MSI-H subtypes is expected using two new biomarkers, *ARID1A* and *ZFHX4*, which account for a high proportion of Chinese EC patients.

## Electronic supplementary material

Below is the link to the electronic supplementary material.

Supplementary Material 1

Supplementary Material 2

Supplementary Material 3

Supplementary Material 4

## Data Availability

The datasets generated and/or analyzed in the current study are available upon reasonable request to the corresponding author.
