# Supplementary figures and images for: Molecular subtyping of endometrial cancer via a simplified one-step NGS classifier, ARID1A and ZFHX4 mutations help further subclassify CNL/MSI-H patients
Source: Diagn Pathol. 2025 Apr 25;20:52. doi: 10.1186/s13000-025-01652-z (PMC12023587; doi:10.1186/s13000-025-01652-z)

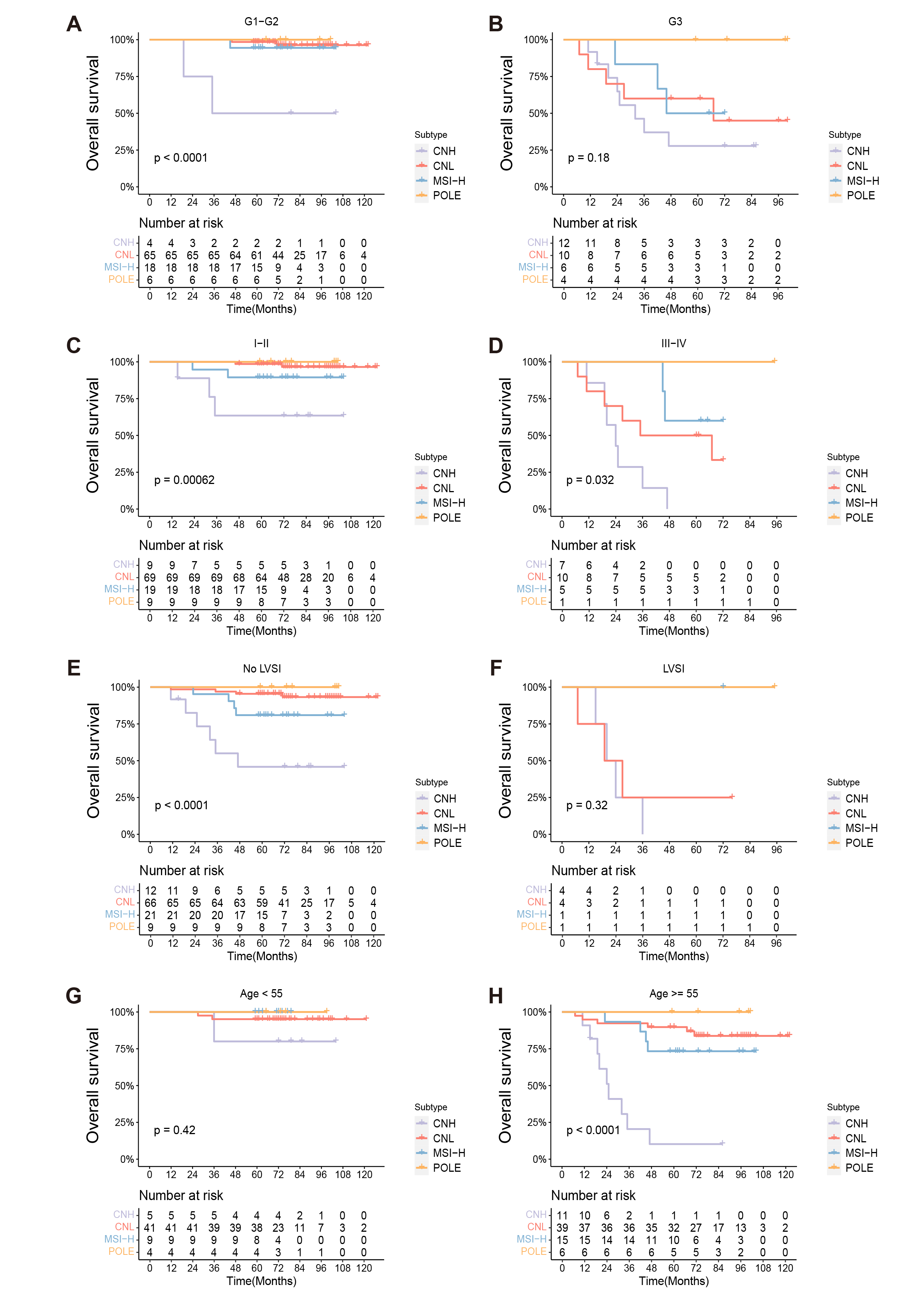

Supplement: Supplementary file 2 — Supplementary Material 2 [file 13000_2025_1652_MOESM2_ESM.tif]
