# Supplementary material for: Molecular subtyping of endometrial cancer via a simplified one-step NGS classifier, ARID1A and ZFHX4 mutations help further subclassify CNL/MSI-H patients
Source: Diagn Pathol. 2025 Apr 25;20:52. doi: 10.1186/s13000-025-01652-z (PMC12023587; doi:10.1186/s13000-025-01652-z)
Supplement: Supplementary file 3 — Supplementary Material 3 [file 13000_2025_1652_MOESM3_ESM.doc]

**Supplementary Table 1.** The main mutation counts (exon5-exon10) in TP53 between the current study and the TCGA cohort.

| **EXON** | **PRESENT (n = 233)** | **TCGA (n = 232)** |
| --- | --- | --- |
| **Exon4** | 4 | 2 |
| **Exon5** | 7 | 13 |
| **Exon6** | 8 | 10 |
| **Exon7** | 7 | 27 |
| **Exon8** | 14 | 17 |
| **Exon9** | 0 | 1 |
| **Exon10** | 0 | 2 |

**Supplementary Table 2.** The main mutation site (exon5-exon8) in TP53 between the current study and the TCGA cohort, which has more than two cases.

| **Exon** | **SITE** | **PRESENT** | **TCGA** |
| --- | --- | --- | --- |
| **Exon5** | R158C | 0 | 2 |
|  | R175H | 2 | 2 |
| **Exon6** | R213* | 2 | 2 |
|  | Y220C | 0 | 3 |
| **Exon7** | S240G | 0 | 2 |
|  | G244S | 0 | 2 |
|  | R248W | 0 | 5 |
|  | R248Q | 2 | 4 |
| **Exon8** | R273H | 5 | 4 |
|  | R273C | 2 | 3 |
