# Supplementary material for: Molecular subtyping of endometrial cancer via a simplified one-step NGS classifier, ARID1A and ZFHX4 mutations help further subclassify CNL/MSI-H patients
Source: Diagn Pathol. 2025 Apr 25;20:52. doi: 10.1186/s13000-025-01652-z (PMC12023587; doi:10.1186/s13000-025-01652-z)
Supplement: Supplementary file 4 — Supplementary Material 4 [file 13000_2025_1652_MOESM4_ESM.docx]

**Supplementary Materials**

**Supplementary figure legends**

**Supplementary Fig. 1**. Comparison of mutant profiles of Key genes in EC. **(A)** 3 Lollipop plot showing the distribution of identified hotspot mutations of 4 POLE gene. **(B)** Lollipop plot showing the distribution of identified hotspot 5 mutations of TP53 gene. **(C)** Lollipop plot showing the distribution of 6 identified hotspot mutations of ARID1A gene. **(D)** Lollipop plot showing the distribution of identified hotspot mutations of ZFHX4 gene.

**Supplementary Fig. 2**. The 10-year overall survival of different molecular subtypes. Overall survival analysis of the four molecular subtypes detected by NGS, patients were stratified according to the grade 1/2 **(A)** or grade 3 **(B)**, clinical stage I-II **(C)** or stage III-IV **(D)**, without LVSI **(E)** or with LVSI **(F)**, and age < 55 **(G)** or age ≥ 55 **(H)**.

**Supplementary table legends**

**Supplementary Table 1.** The main mutation counts (exon5-exon10) in TP53 between the current study and the TCGA cohort.

**Supplementary Table 2.** The main mutation site (exon5-exon8) in TP53 between the current study and the TCGA cohort, which has more than two cases.
